# Vitamins in the Treatment of Periodontitis: Molecular Mechanism and Network Pharmacological Analysis

**DOI:** 10.3290/j.ohpd.c_2384

**Published:** 2025-12-12

**Authors:** Lu-Ran Wang, Dong-Xu Liu, Lei Yu, Li Gao

**Affiliations:** a Lu-Ran Wang Dentist, Department of Oral and Maxillofacial Surgery, The Second Affiliated Hospital of Harbin Medical University, Harbin, China. Writing – original draft, visualisation, writing – review and editing, methodology, investigation, data curation, and conceptualisation.; b Dong-Xu Liu Dentist, Department of Oral and Maxillofacial Surgery, The Second Affiliated Hospital of Harbin Medical University, Harbin, China. Writing – original draft, visualisation, data curation, and conceptualisation.; c Lei Yu Dentist, Department of Colorectal Surgery, The Second Affiliated Hospital of Harbin Medical University, Harbin, China. Writing – review and editing, writing – original draft, supervision, project administration, investigation, and conceptualisation.; d Li Gao Dentist, Department of Oral and Maxillofacial Surgery, The Second Affiliated Hospital of Harbin Medical University, Harbin, China. Writing – review and editing, writing – original draft, supervision, project administration, investigation, and conceptualisation.

**Keywords:** anti-inflammatory mechanisms, antioxidant therapy, bone metabolism, immune regulation, network pharmacology, oxidative stress, periodontitis, vitamins.

## Abstract

**Purpose:**

Periodontitis manifests as a chronic inflammatory condition triggered by pathogenic microbial colonisation, and its pathogenesis involves the interaction of multiple factors such as oxidative stress, immune imbalance and abnormal bone metabolism. In recent years, the role of nutritional factors, especially vitamins, in regulating inflammation, oxidative stress and bone regeneration has gradually attracted attention, but their specific mechanisms and clinical application potential still need to be systematically explored. We hypothesised that vitamins A, C, D, E, and K confer protection against periodontitis by synergistically modulating a shared network of targets central to inflammatory and bone metabolic pathways.

**Material and Methods:**

This study is a review that includes computer simulation analysis. The objective of this research is to analyse the role of vitamins (D, C, E, A, and K) in the prevention and management of periodontitis, and to reveal their potential molecular targets through network pharmacological analysis, so as to provide a theoretical basis for the clinical application of vitamins.

**Results:**

Studies have shown that vitamins affect periodontal health in a number of ways: anti-inflammatory and antioxidant effects; vitamin C and E reduce oxidative stress by scavenging reactive oxygen species (ROS) and by inhibiting the release of inflammatory factors; vitamin D regulates immune cell function and reduces the production of pro-inflammatory mediators. Regulation of bone metabolism: vitamins D, K, and A can improve alveolar bone resorption by promoting osteoblast differentiation and inhibiting osteoclast activity. Network pharmacology analysis further screened out the core targets and verified their association with inflammation and bone metabolism pathways through molecular docking. Clinical studies have shown that vitamin supplementation can significantly improve periodontal probing depth, loss of attachment, and bleeding index.

**Conclusions:**

Vitamins have multiple potentials in the prevention and treatment of periodontitis, such as anti-inflammatory, antioxidant and bone regeneration, but their clinical application needs to consider individual differences, bioavailability and synergistic effects. Vitamins D and C emerge as the most critically supported micronutrients for adjunctive periodontitis management. For clinical practitioners, ensuring patients’ adequacy in vitamin D is paramount for its immunomodulatory and bone-stabilising benefits, while vitamin C supplementation is strongly indicated for its antioxidant capacity and role in collagen synthesis. While vitamins K, A, and E show promising mechanistic roles, their routine supplementation requires more targeted evidence. Clinical application must consider individual nutritional status, bioavailability, and synergistic effects within a precision nutrition strategy. In the future, it is necessary to combine precision nutrition strategies and clinical validation to promote the transformation of vitamins from basic research to personalised treatment.

Periodontitis, designated as a prevalent chronic inflammatory malady across the globe,^35^ exerts a comprehensive impact on a diverse array of tissues, with a predominant focus on the structural components that underpin dental stability (Fig 1). This encompasses the gums, periodontal ligaments, alveolar bone, and cementum, all of which are integral to the maintenance of dental integrity. The inexorable progression of periodontitis culminates in a gradual, insidious loosening of the affected tooth, ultimately culminating in its premature attrition.^3^ And in severe cases, periodontitis can also adversely affect the overall health of the patient. As periodontitis plays a core role in the association between oral and maxillofacial diseases and systemic diseases, it has been proven to be a condition with major systemic impacts. It may even affect the development of cancer by influencing the tumour microenvironment.^65^ Therefore, research on overall and individualised health approaches for periodontitis is also on the rise.^32^ Periodontitis is also increasingly regarded as an independent cardiovascular risk factor.^11^ It has also been proven to be potentially related to neurological diseases such as Alzheimer’s disease.^13^ The pathogenesis of periodontitis is extremely complex, involving the interaction of multiple factors such as smoking, oral hygiene, microbial infection, host immune response, and genetic factors.^26,48^ However, it is undeniable that nutritional factors are also very important factors in the process of causing the systemic inflammatory response.^20,22^ Studies have shown that dietary habits and nutrition have a significant impact on oral health.^7,43^ The prevention, early diagnosis and targeted intervention of periodontitis are important parts of public health.^57^

**Fig 1:**
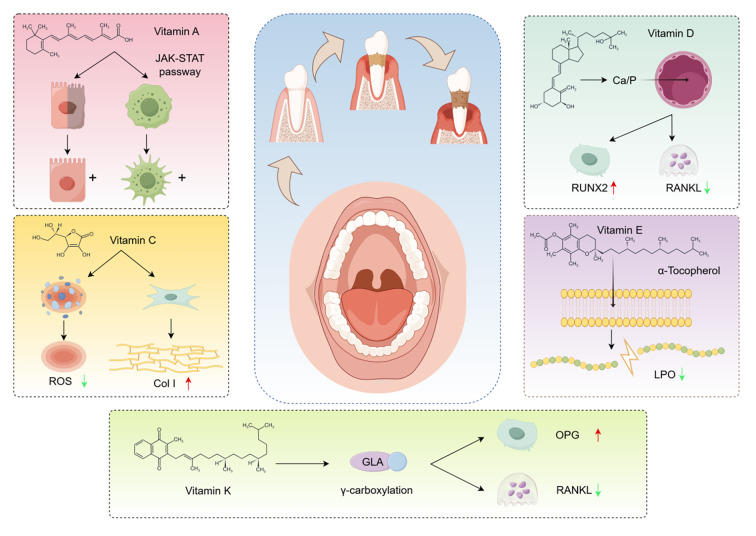
The multifaceted mechanism of action of vitamins.

## Vitamins and Inflammation of the Oral Cavity

In recent years, the role of vitamins in maintaining oral health has gradually become a research hotspot. Vitamins, being crucial nutrients, are necessary for maintaining the body’s normal physiological operations. In the prevention and treatment of periodontitis, the role of vitamins cannot be ignored. Studies have shown that vitamins have significant antioxidative stress properties and can effectively exert anti-inflammatory effects,^16^ and oxidative stress is considered to be one of the important pathogenic mechanisms of periodontitis.^15^ Therefore, the potential value of vitamins in the treatment of periodontitis is worth exploring and exploiting.

In the treatment of periodontitis, the anti-inflammatory, antioxidant and bone-promoting effects of vitamins are increasingly emphasised. Periodontitis is a long-term inflammatory condition triggered by a microbial infection, resulting in the destruction of gum tissue and alveolar bone. Studies have shown that vitamin A, vitamin D, vitamin C, vitamin E and vitamin K play an important role in regulating immunity, resisting oxidative stress and promoting bone metabolism.

At first, the attention given to vitamin D’s effects on periodontal health has been considerable. Vitamin D promotes bone health by regulating the metabolism of calcium and phosphorus, and its active form, 1,25-dihydroxyvitamin D3, has been found to inhibit the growth of bacteria associated with periodontitis and reduce inflammatory responses.^54^ In addition, vitamin D also plays an important role in maintaining periodontal health by inducing the expression of the antimicrobial peptide LL-37 and enhancing the antimicrobial ability of oral epithelial cells.^38^ Further studies have shown that vitamin D deficiency is closely associated with the occurrence of periodontitis, and its supplementation may have a positive effect on reducing the symptoms of periodontitis and promoting alveolar bone regeneration.^8^

In addition to vitamin D, vitamin C and vitamin A have also shown potential benefits in the management of periodontitis. Vitamin C acts as a powerful antioxidant, neutralising free radicals and reducing oxidative stress damage to periodontal tissues. Studies have found that vitamin C intake is negatively correlated with the risk of periodontitis, and higher vitamin C levels can significantly reduce the incidence of periodontitis.^41^ In addition, vitamin C plays a key role in the synthesis of collagen, promoting the repair and regeneration of periodontal tissues. Vitamin C supplementation can improve bleeding gums and other periodontitis symptoms, suggesting the importance of vitamin C in maintaining periodontal health.^39,64^ Vitamin A is considered to potentially benefit periodontal health, but the evidence is disputed, requiring additional research to elucidate the exact mechanism.^41^

Vitamin E is crucial for its anti-inflammatory and antioxidant properties. Research indicates that consuming vitamin E is linked to a lower risk of periodontitis, and moderate consumption can decrease the likelihood of developing the condition.^38,64^ Vitamin E attenuates the inflammatory response of periodontal tissues by inhibiting the release of inflammatory mediators, thereby contributing to bone health and periodontal stability.

In summary, vitamins have a significant role in anti-inflammatory, antioxidant and bone formation promotion in periodontitis. Vitamin supplementation through a reasonable diet can not only enhance the body’s immune function but also effectively reduce the risk of periodontitis and improve oral health. Further studies should investigate the combined effects of various vitamins and their potential use in treating periodontitis to offer new strategies and insights for its prevention and treatment.

## Network Pharmacology and Vitamins

Network pharmacology is a multidisciplinary research approach that integrates bioinformatics, computational biology, and network science with the aim of revealing the complex relationships between drugs and their targets by constructing networks of interactions between biomolecules. In the study of vitamins and periodontitis, network pharmacology has been widely used to explore the mechanism of action of vitamins and their potential applications in the treatment of periodontitis. By integrating existing bioinformation and clinical data, network pharmacology provides a new perspective and theoretical basis for the application of vitamins in the treatment of periodontitis. The application of network pharmacology has enabled researchers to more fully understand the multi-target role of vitamins in the treatment of periodontitis. By constructing a network of vitamins and periodontitis-related targets, researchers can identify key biological pathways that affect periodontal health, thereby providing new therapeutic strategies for clinical practice. This approach not only helps to identify potential biomarkers but also provides a theoretical basis for individualised treatment and facilitates the use of vitamin supplementation in the management of periodontitis.

In summary, network pharmacology provides a new perspective on the multi-target role of vitamins in the treatment of periodontitis, and through systematic analysis and integration, it can provide important guidance for future research and clinical practice, especially in the development of new treatments and optimisation of existing treatment options.

## The Multifaceted Role and Mechanism of Vitamins

The role of vitamins is vital for maintaining health, particularly in the prevention and management of periodontitis. Different vitamins, such as vitamins D, C, A, and K, exhibit their own unique mechanisms in immune regulation, anti-inflammatory effects, and bone metabolism, affecting multiple aspects of periodontal health.

To begin with, vitamin D is important for the regulation of immunity and bone health. Studies have shown that the active form of vitamin D, 1,25(OH)2D3, is able to promote the expression of antimicrobial peptides and inhibit the growth of bacteria associated with periodontitis by regulating the activity of immune cells.^25^ Vitamin D deficiency is also inversely linked to the severity of chronic periodontitis, and supplementing with vitamin D is believed to enhance periodontal health and facilitate bone remodelling.^40^ Moreover, a lack of vitamin D is negatively associated with the severity of chronic periodontitis, and taking vitamin D supplements is believed to enhance periodontal health and aid in bone remodelling.

Secondly, vitamin C has been extensively studied for its role in periodontal health due to its powerful antioxidant properties and ability to promote collagen synthesis. Systematic reviews have shown that vitamin C supplementation is associated with a reduced risk of periodontal disease, and that its protective effect against periodontitis may be related to its ability to reduce inflammation and promote gum tissue repair.^9^ Vitamin C not only improves the health of periodontal tissues but may also defend against the progression of periodontitis by enhancing the host’s immune response.

The role of vitamin A in the immune system is important, as it impacts the maintenance of epithelial cells and the regulation of immune responses. Studies have found that vitamin A insufficiency may be associated with the development of periodontal disease, especially if a strong immune response is required.^41^ This suggests that adequate intake of vitamin A may help maintain the health of periodontal tissues.

Vitamin K is also indispensable in bone metabolism, especially in bone mineralisation and calcium metabolism. It helps maintain bone density and health by activating vitamin K-dependent proteins.^66^ In patients with chronic kidney disease, vitamin K deficiency may exacerbate bone disease, so vitamin K supplementation may help improve bone health.

Finally, the multiple mechanisms of action of vitamins are not only reflected in the function of single vitamins, but also in the interaction between them. For example, vitamin D and vitamin K have a synergistic relationship that is crucial for bone metabolism, aiding in the regulation of bone health and the prevention of osteoporosis.^1^ This synergy provides a new perspective on the prevention and treatment of periodontal disease.

In summary, the multiple mechanisms of action of various vitamins in immunomodulation, anti-inflammation and bone metabolism provide new ideas for the prevention and treatment of periodontitis. Based on the established but fragmented evidence regarding the anti-inflammatory, antioxidant, and immunomodulatory properties of vitamins, we propose a unifying hypothesis. We hypothesised that the therapeutic potential of vitamins A, C, D, E, and K in periodontitis stems from their ability to concurrently target multiple nodes within the complex network of periodontal pathogenesis, despite their distinct chemical structures. Specifically, we postulated that network pharmacology analysis would reveal a set of common high-value targets (eg, key transcriptional regulators and inflammatory mediators) shared across multiple vitamins, and that molecular docking would confirm their strong binding affinity, thereby providing a mechanistic basis for their adjunctive use. Future research should further explore the specific application and potential mechanism of vitamin supplementation in periodontal health to provide a theoretical basis and practical guidance for the effective treatment of periodontitis.

## Methodology

A total of 3,639 periodontitis-related targets were screened out from the Genecards database (www.genecards.org). In addition, 2,016 inflammatory targets, 2,001 oxidative stress targets, and 3,149 bone formation targets were screened out. The target of periodontitis intersected with three mechanism targets, yielding 603 genes as its mechanism targets. A total of 100 targets each for vitamins A, C, D, E and K were obtained from the SwissTargetPrediction platform (www.swisstargetprediction.ch). The targets of these vitamins were respectively intersected with 603 mechanism targets of periodontitis, resulting in 24 intersection targets for vitamin A and 14 intersection targets for vitamin C. There are 17, 29, and 23 vitamins D, E, and K, respectively. Build PPI (protein–protein interaction) networks using STRING databases, respectively and remove isolated nodes. These genes were respectively placed into the Cytoscape software for cytohubba and MCODE (Molecular Complex Detection) analysis to screen out the top 10 core targets in terms of degree value ranking. Download the 3D structure of small molecules from the Pubchem website (pubchem.ncbi.nlm.nih.gov), download the 3D structure of proteins from the UniProt database (www.uniprot.org), and perform molecular docking using AutoDock Vina.

## The Role of Vitamins in Periodontitis

### The role of vitamin A

Vitamin A, also known as retinol, is a key nutrient for maintaining mucosal barrier integrity and immune function,^56^ a crucial dietary element for humans, important for vision preservation, embryonic growth, immune system enhancement, and tissue repair and homeostasis.^47^ Vitamin A exerts different effects on various stages of the osteogenic process through the regulation of retinoic acid receptor (RAR) signalling and osteoblast/osteoblast-associated octapeptides.^63^ Vitamin A affects the function of immune cells through its metabolite retinol. Studies have found that vitamin A can promote the development of T cells and enhance the body’s immune response to bacterial infections,^38^ enhancing the host’s ability to clear pathogenic microorganisms. Vitamin A is essential for both mucosal integrity and the proper functioning of the immune system.^28^ Vitamin A deficiency may lead to gingival keratosis, decreased saliva production, and weakened immunity. Research indicates that appropriate vitamin A supplementation improves clinical markers of periodontal disease, as evidenced by reduced probing depths and attenuated attachment loss.^58^

### Mechanism of vitamin A in the treatment of periodontitis

Research on clinical micronutrient consumption indicates that adequate intake of vitamins A and B2 is linked to a lower occurrence of periodontitis.^37^ At the same time, studies have shown that there is a negative correlation between the consumption of high-dose vitamins, vitamin B complex, vitamin C, vitamin D and vitamin E and the likelihood of periodontal disease,^44^ and some studies have shown that dietary consumption of vitamin A has a certain correlation with the occurrence and development of periodontal disease,^30^ suggesting that vitamins can play an important role in the prevention of periodontal disease. An *in vitro* study found that vitamin A exerts a therapeutic role in periodontitis by regulating the inflammatory response through the JAK-STAT pathway, particularly by influencing metabolic reprogramming of macrophages.^18^ The role of vitamin A in immunomodulation is achieved by enhancing the antimicrobial capacity of epithelial cells. The active form of vitamin A (retinol) stimulates the growth and differentiation of oral epithelial cells and promotes the expression of antimicrobial peptides such as LL-37, which help fight bacterial infections and reduce the incidence of periodontitis.^17^ In addition, vitamin A is involved in the regulation of bone metabolism, which is essential for the health of periodontal support tissues. Studies have found that vitamin A regulates the function of osteoblasts and promotes bone regeneration, while observational studies have shown that periodontitis is often accompanied by increased bone resorption.^38^

**Fig 2a to e:**
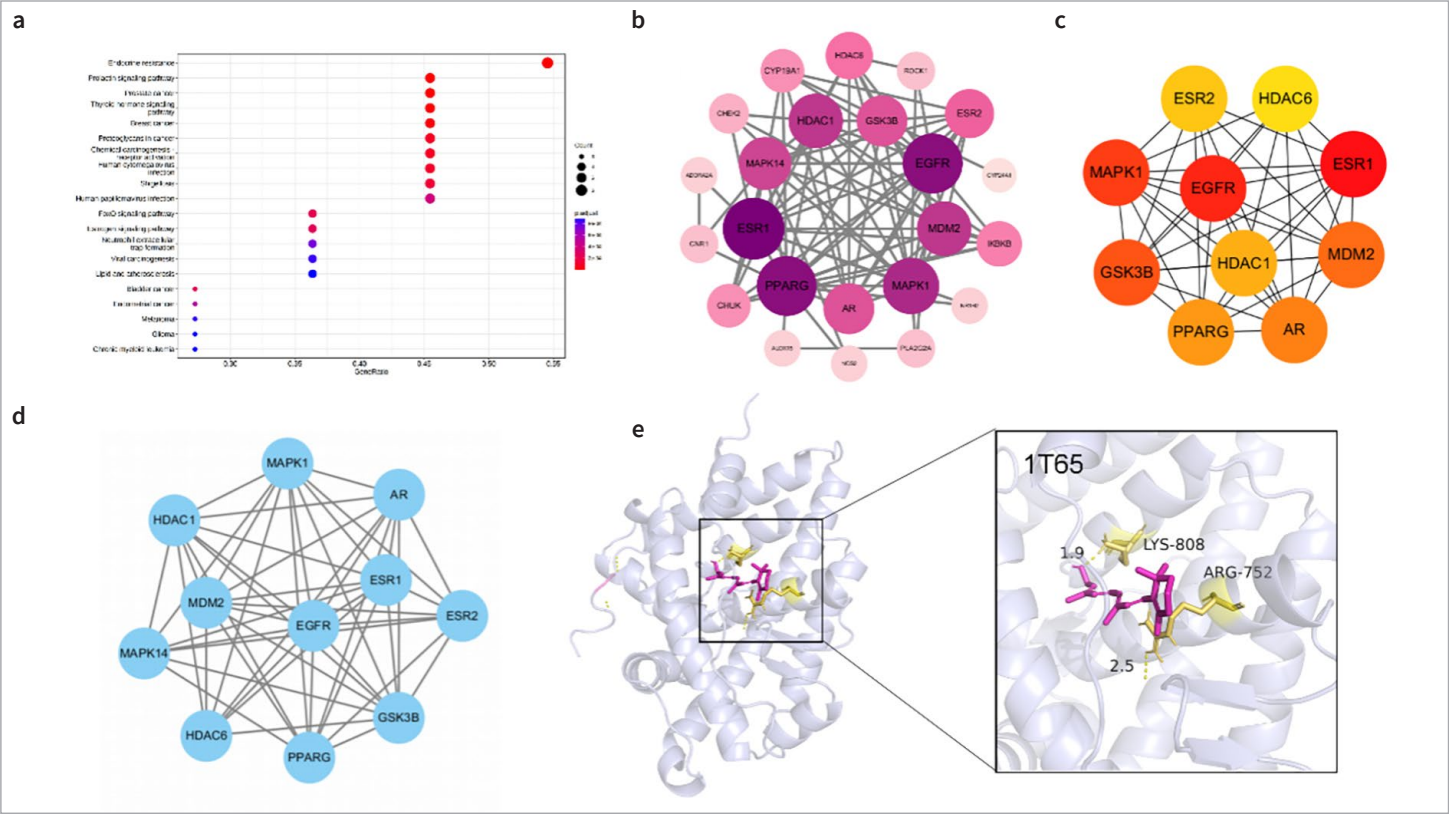
Network pharmacologic results of vitamin A treatment for periodontitis. (a) Vitamin A and periodontitis disease target KEGG enrichment analysis chart. (b) Interaction map for vitamin A and periodontitis disease targets. Arrange them in descending order of degree value and from deep to shallow. (c) Vitamin A and periodontitis Hubba gene disease targets. (d) Mcode classification of vitamin A and periodontitis disease targets.(e) Molecular docking results of vitamin A and periodontitis disease targets. The molecular docking results indicated the binding affinity between vitamin A and the core target protein (PDB:1T65) and marked the key residues.

The GeneCards database yielded 100 potential vitamin A targets. Intersecting these with 603 known periodontitis-associated genes identified 24 common targets, which were subsequently used to construct a PPI network using the STRING platform (Fig 2b). Application of the Molecular Complexity Detection (MCODE) algorithm pinpointed densely interconnected network modules. Finally, 10 high-probability core targets were selected based on their DEGREE scores: *ESR1*, *EGFR*, *MAPK1*, *GSK3B*, *MDM2*, *AR*, *PPARG*, *HDAC1*, *ESR2*, and *HDAC6* (Fig 2c). Molecular docking is performed. Notably, we selected 1T65 for molecular docking and found that vitamin A mainly interacted with the target of 1T65 at the positions 808, LYS and 752, ARG residues (Fig 2e). The core targets were analysed by KEGG Kyoto Encyclopedia of Genes and Genomes pathway enrichment (Fig 2a).

### The role of vitamin C

Vitamin C, or ascorbic acid, is essential for preserving human health, but it cannot be synthesised by the body itself, only through food intake.^49^ Having insufficient vitamin C can result in health issues, including bleeding gums and impaired immune defences, making the body more susceptible to infections.^10^ Vitamin C is involved in the synthesis of collagen and is an important factor in providing structural strength to connective tissue.^51^ Vitamin C deficiency, weak collagen fibres and connective tissue structure, difficult wound healing, loose teeth, weakened immunity, and increased risk of serious infections such as pneumonia.^29^ Several studies have confirmed that vitamin C has significant antioxidant properties, it can quickly remove oxidative free radicals in the body, and maintain the homeostasis of redox reactions,^4^ which is essential for the normal physiological activities of cells. More seriously, long-term vitamin C deficiency can also induce scurvy,^14^ which can have serious health effects.

### Mechanism of vitamin C in the treatment of periodontitis

Vitamin C plays a vital role in enhancing the antioxidant defence mechanism of gum tissue, effectively alleviating oxidative stress by significantly reducing the amount of reactive oxygen species (ROS) within the tissue.^15^ Numerous studies have consistently shown that maintaining physiologically low ROS levels of antioxidant status is considered to be a key factor in attenuating tissue damage caused by periodontitis and its disease progression.^24^ In addition, clinical evidence has shown that vitamin C supplementation can significantly improve the bleeding index in gingivitis patients, further confirming the positive role of vitamin C in periodontal health maintenance.^23^ In addition, *in vitro* study has been shown to slow the rate of progression of periodontitis by stimulating the differentiation of periodontal ligament progenitor cells.^61^ Clinical research has shown that vitamin C supplementation can improve postoperative recovery and reduce the inflammatory response in periodontal tissues. This finding further highlights the value of vitamin C as a potential adjunctive therapy, especially when performing non-surgical periodontal treatment.^46^ In addition, vitamin C has been found to inhibit periodontitis-related inflammatory markers, such as C-reactive protein (CRP) and cytokines,^62^ and reducing these factors helps reduce the inflammatory response in periodontal tissue. Not only that, but vitamin C also plays an important role in bone metabolism. In some clinical experiments, vitamin C supplementation has been found to improve the structure of alveolar bone and inhibit bone resorption, thereby reversing bone loss due to periodontitis to some extent.^28^

**Fig 3a to e:**
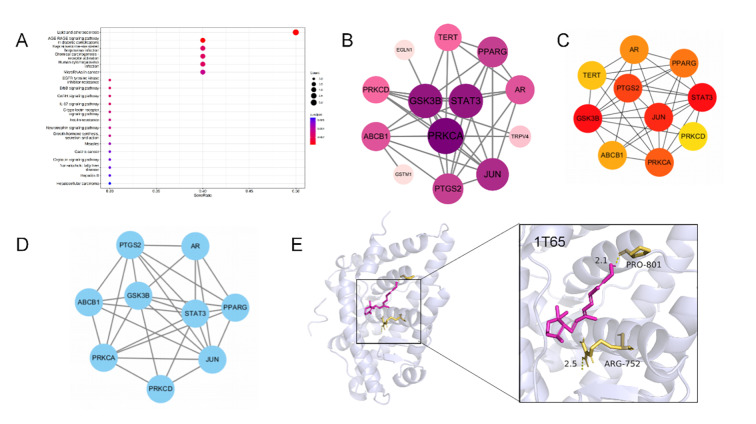
Network pharmacologic results of vitamin C treatment for periodontitis. (a) Vitamin C and periodontitis disease target KEGG enrichment analysis chart. (b) Interaction map for vitamin C and periodontitis disease targets. Arrange them in descending order of degree value and from deep to shallow. (c) Vitamin C and periodontitis Hubba gene disease targets. (d) Mcode classification of vitamin C and periodontitis disease targets. (e) Molecular docking results of vitamin C and periodontitis disease targets. The molecular docking results indicated the binding affinity between vitamin C and the core target protein (PDB:1T65) and marked the key residues.

A total of 3,639 targets related to periodontitis, 3,149 targets related to bone formation, 2,001 targets associated with oxidative stress, and a total of 2,016 targets associated with inflammation were obtained from the database. The targets of periodontitis were intersected with the three main types of pathogenic factors, and 603 mechanistic gene targets of periodontitis were obtained. These 603 mechanistic gene targets intersected with 45 vitamin C-related targets collected in the database, and 14 common targets were obtained. These common targets of periodontitis and vitamin C were used to construct PPI networks in the STRING platform (Fig 3b), and subsequently, the Molecular Complexity Detection (MCODE) algorithm pinpointed densely interconnected network modules; 10 high-probability core targets were subsequently selected based on their DEGREE scores – *STAT3*, *GSK3B*, *JUN*, *PTGS2*, *PRKCA*, *PPARG*, *AR*, *ABCB1*, *TERT*, and *PRKCD* – were selected (Fig 3c), and molecular docking was performed. It is worth noting that through molecular docking with 1T65, it was found that vitamin C mainly interacted with the target at 801, PRO and 752, ARG of the 1T65 to regulate its function (Fig 3e). The selected core targets were also used for KEGG pathway enrichment analysis (Fig 3a).

### The role of vitamin D

Vitamin D is a fat-soluble vitamin that is essential for human health. It is a major factor in intestinal calcium absorption,^19^ can promote the absorption of calcium and phosphorus, contribute to the formation and maintenance of bones, and is a key factor in regulating calcium and phosphorus homeostasis and mineral bone metabolism.^53^ Vitamin D can regulate the function of the immune system, promote the production of anti-inflammatory cytokines, and inhibit the release of pro-inflammatory cytokines, thereby reducing the inflammatory response,^36,58^ helping to prevent inflammatory diseases. Vitamin D, acting through its active form 1,25(OH)2D3, can activate autophagy, which in turn enhances host cell resistance to pathogens and reduces inflammatory responses.^17^ In addition, studies have shown that vitamin D can affect the metabolism of osteoclasts, thereby reducing bone resorption, maintaining bone health, and preventing fractures and osteoporosis.^60^

Vitamin D deficiency may affect the normal growth and maintenance of bones, which may lead to diseases such as rickets and osteoporosis.

### Mechanism of vitamin D in the treatment of periodontitis

Vitamin D inhibits the activation of inflammatory cells and reduces the production of inflammatory factors, thereby reducing the inflammatory response of periodontal tissues. Vitamin D contributes to the absorption and utilisation of calcium and phosphorus, promotes bone regeneration of periodontal tissues, and helps repair damaged periodontal tissues.^39,52^ Clinical studies have shown that, serum vitamin D levels have been shown to be lower in patients with periodontitis than in healthy controls.^42^ Vitamin D has a certain antibacterial effect, which can inhibit the growth of periodontal disease-related bacteria and reduce the occurrence and progression of periodontal disease. In addition, it has been found in clinical trials that patients with higher serum vitamin D levels show less bleeding on probing, better probing depth, less loss of clinical attachment, less tooth loss, and lower pathogenic levels,^2^ all suggesting the potential therapeutic role of vitamin D in periodontitis. Several studies have shown that the combination of vitamin D supplementation with non-surgical periodontal therapy can significantly improve periodontal health and clinical indicators such as probing depth and clinical attachment loss.^42^

**Fig 4a to e:**
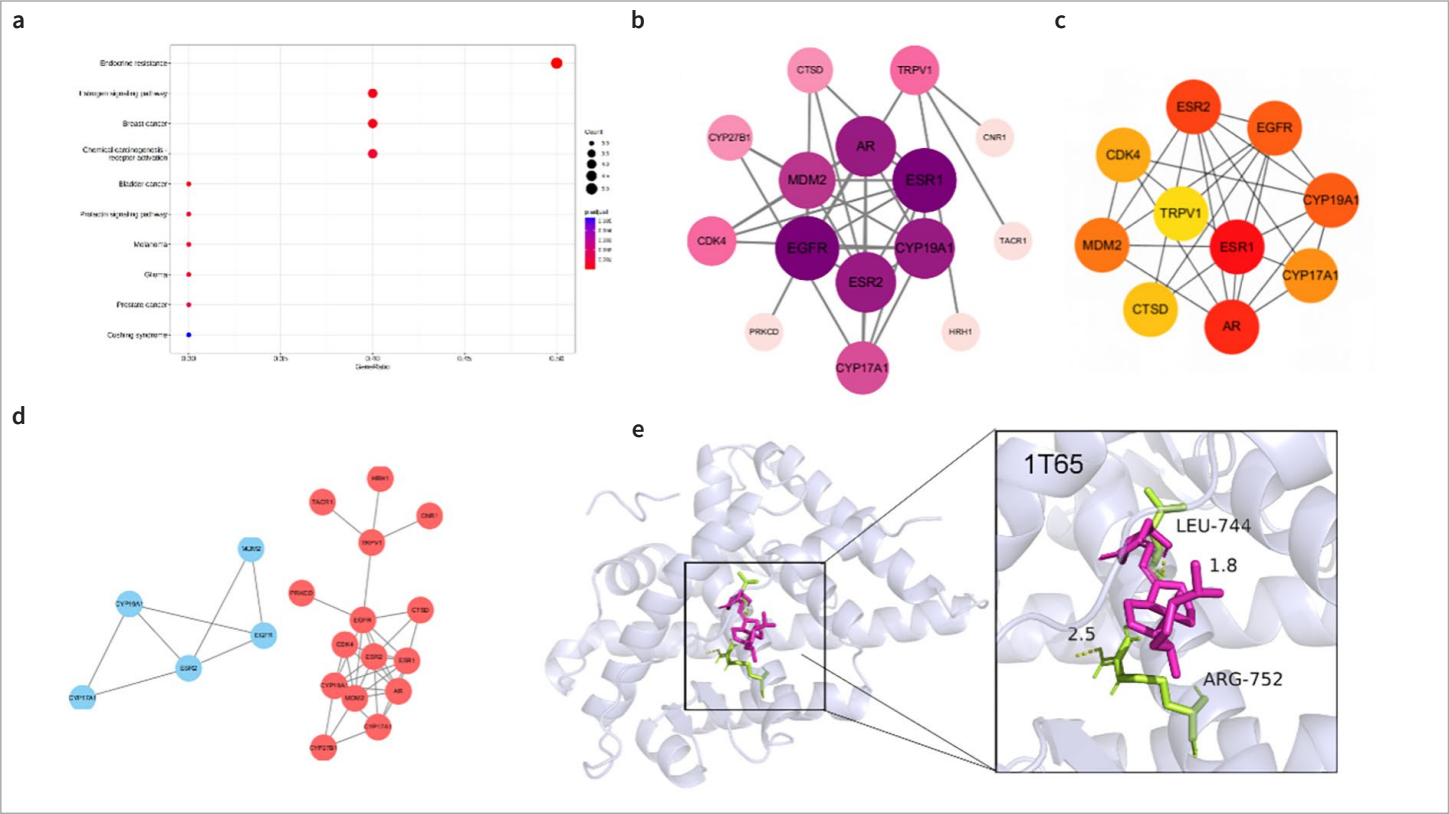
Network pharmacologic results of vitamin D treatment for periodontitis.(a) Vitamin D and periodontitis disease target KEGG enrichment analysis chart. (b)Interaction map for vitamin D and periodontitis disease targets. Arrange them in descending order of degree value and from deep to shallow. (c) Vitamin D and periodontitis Hubba gene disease targets. (d) Mcode classification of vitamin D and periodontitis disease targets. (e) Molecular docking results of vitamin D and periodontitis disease targets. The molecular docking results indicated the binding affinity between vitamin D and the core target protein (PDB:1T65) and marked the key residues.

A total of 100 vitamin D-related targets were screened from the database and intersected with 603 periodontitis mechanism gene targets, and a total of 17 targets were integrated into the STRING platform to establish a PPI network (Fig 4b). By utilising the list of screened genes, the more tightly connected network components were identified by the Molecular Complexity Assay (MCODE) algorithm. 10 core targets with high degree values, including *ESR1*, *AR*, *ESR2*, *EGFR*, *CYP19A1*, *MDM2*, *CYP17A1*, *CDK4*, *CTSD*, *TRPV1* (Fig 4c), were screened for molecular docking. Notably, 1T65 was chosen for molecular docking analysis. This analysis showed that vitamin D mainly interacted with LEU at position 744 of 1T65 target and ARG at position 752 (Fig 4e). The core targets were used for KEGG pathway enrichment analysis (Fig 4a).

### The role of vitamin E

Vitamin E is a class of fat-soluble antioxidants, mainly tocopherols and tocotrienols, whose core function is to protect cell membranes from oxidative damage. According to some studies, taking vitamin E supplements may help prevent cardiovascular disease (CVD) by acting as an antioxidant, preventing the oxidation of lipoproteins and avoiding platelet aggregation.^55^ In addition, epidemiological studies have shown that vitamin E has also been linked to neurodegenerative diseases.^31^ As an important free radical scavenger, vitamin E can interrupt the lipid peroxidation chain reaction and maintain the structural integrity of cell membranes. Studies have shown that vitamin E has powerful anti-inflammatory properties and therefore strengthens the immune system.^12^ Studies have shown that vitamin E deficiency may lead to increased oxidative stress, triggering tissue inflammation and repair dysfunction.

### Mechanism of vitamin E in the treatment of periodontitis

Vitamin E has a powerful antioxidant capacity. Studies have shown that vitamin E can significantly reduce the level 27456681 of reactive oxygen species (ROS) in periodontal tissues, effectively degrade hydroxyl radicals, and further reduce cell oxidative damage, thereby reducing oxidative stress damage to gums and alveolar bone.^59^ *In vitro *experiments have shown that vitamin E also reduces inflammation and promotes tissue repair by inhibiting the release of inflammatory mediators such as interleukins.^59^ Some dietary surveys have shown that fruits and vegetables with higher vitamin E content are negatively associated with the risk of periodontal disease, and vitamin E supplementation combined with scaling and root planing (SRP) treatment can reduce the inflammatory process of periodontitis, improve periodontal clinical indicators, and reduce attachment loss,^6^ and some clinical studies have shown that higher vitamin E intake reduces the risk of periodontitis,^37^ suggesting that vitamin E has a potential disease-modifying effect on periodontitis.

**Fig 5a to e:**
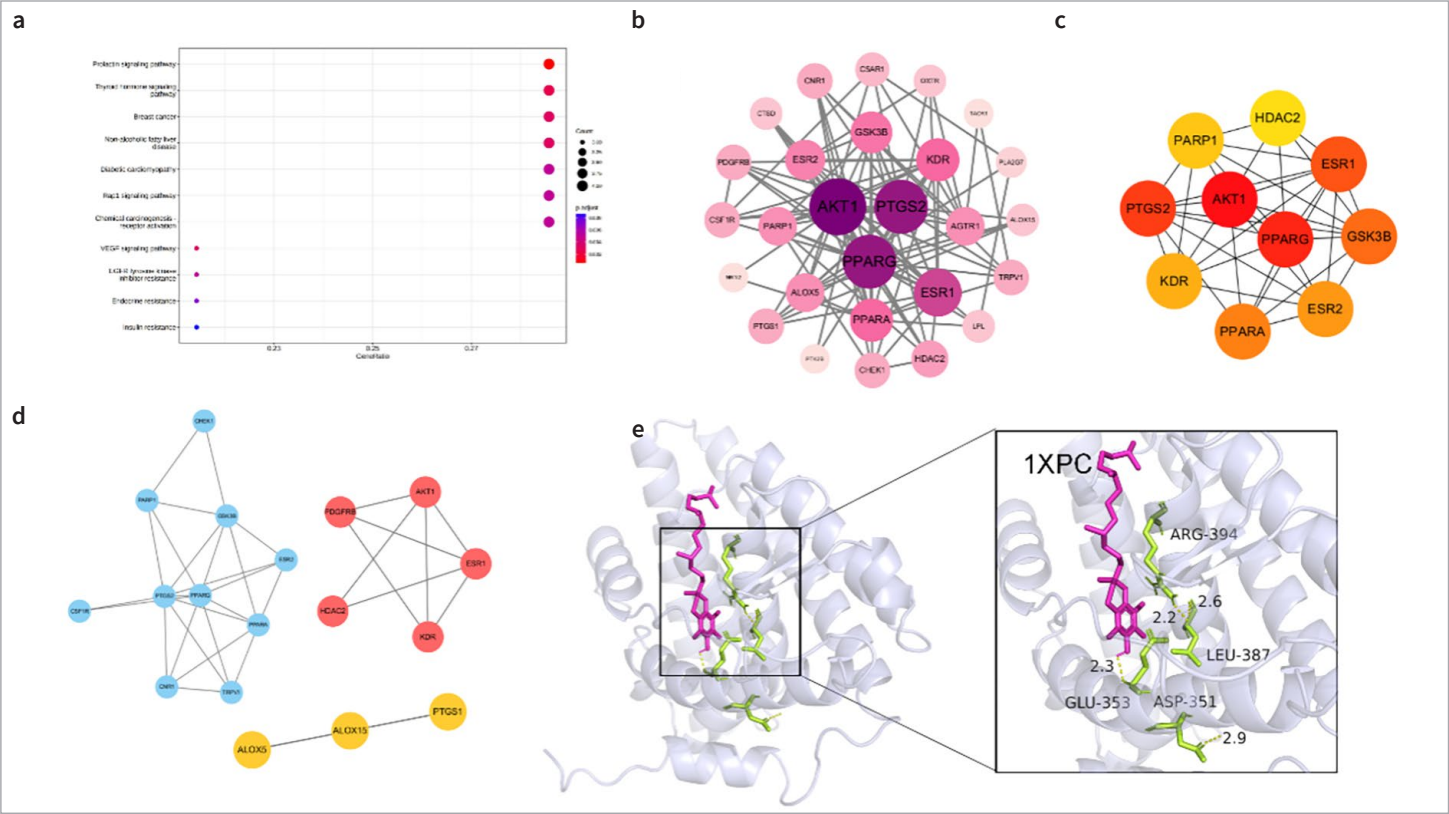
Network pharmacologic results of vitamin E treatment for periodontitis.(a) Vitamin E and periodontitis disease target KEGG enrichment analysis chart. (b) Interaction map for vitamin E and periodontitis disease targets. Arrange them in descending order of degree value and from deep to shallow. (c) Vitamin E and periodontitis Hubba gene disease targets. (d) Mcode classification of vitamin E and periodontitis disease targets. (e) Molecular docking results of vitamin E and periodontitis disease targets. The molecular docking results indicated the binding affinity between vitamin E and the core target protein (PDB:1XPC) and marked the key residues.

A total of 100 vitamin E targets were screened, and 603 targets of periodontitis mechanism genes were intersected with these 100 targets, and a total of 29 common genes were introduced into the STRING platform to construct a PPI network map (Fig 5b). Using the Molecular Complexity Assay (MCODE) algorithm, the more tightly connected network components were identified based on the list of genes found. Then, according to the DEGREE value, the 10 core targets with the highest correlation were selected, namely: *AKT1*, *PPARG*, *PTGS2*, *ESR1*, *GSK3B*, *PPARA*, *ESR2*, *KDR*, *PARP1*, and *HDAC2 *(Fig 5c). Molecular docking is performed. The molecular docking with 1XPC showed that vitamin E mainly interacted with the 394-position ARG target of 1XPC and interacted with the 387-position LEU and 353-position GLU (Fig 5e), thereby affecting its function. The core targets were analysed using KEGG pathway enrichment analysis (Fig 5a).

### The role of vitamin K

Vitamin K is a group of fat-soluble vitamins that include K1 (phylloquinone) and K2 (menaquinone), and vitamin K plays an important role in many physiological processes, especially in bone metabolism and immune regulation. Its core function is to participate in the activation of coagulation factors and the regulation of calcium metabolism.^27^ Vitamin K supplementation may reduce the risk of increased vascular calcification and inflammation.^34^ In addition, vitamin K2 has immunomodulatory effects,^50^ and modulates inflammatory responses, inhibiting the release of pro-inflammatory mediators (such as IL-1β and IL-6) and enhancing the antimicrobial activity of macrophages.^33^

### Mechanism of vitamin K in the treatment of periodontitis

A series of studies has revealed the important role of vitamin K in maintaining gum health. Specifically, vitamin K has a high affinity for the phospholipid region of the cell membrane, and this high binding property contributes to its function during hemostasis.^45^ These mechanisms work together to effectively prevent the occurrence of bleeding gums. At the same time, it has been shown in the literature that vitamin K intake is closely related to its hemostatic effect.^5^ Clinical research has found that inadequate vitamin K intake has been shown to lead to worsening periodontal attachment loss, particularly in the elderly population.^21^ Observational studies have shown that adequate intake of vitamin K has been shown to be associated with increased bone mineral density and may reduce the risk of bone loss due to periodontitis by promoting osteoblast function and inhibiting osteoclast activity.^38^ Therefore, it can be concluded that vitamin K, through the mechanisms described above, plays an important role in the prevention of bleeding gums.

**Fig 6a to e:**
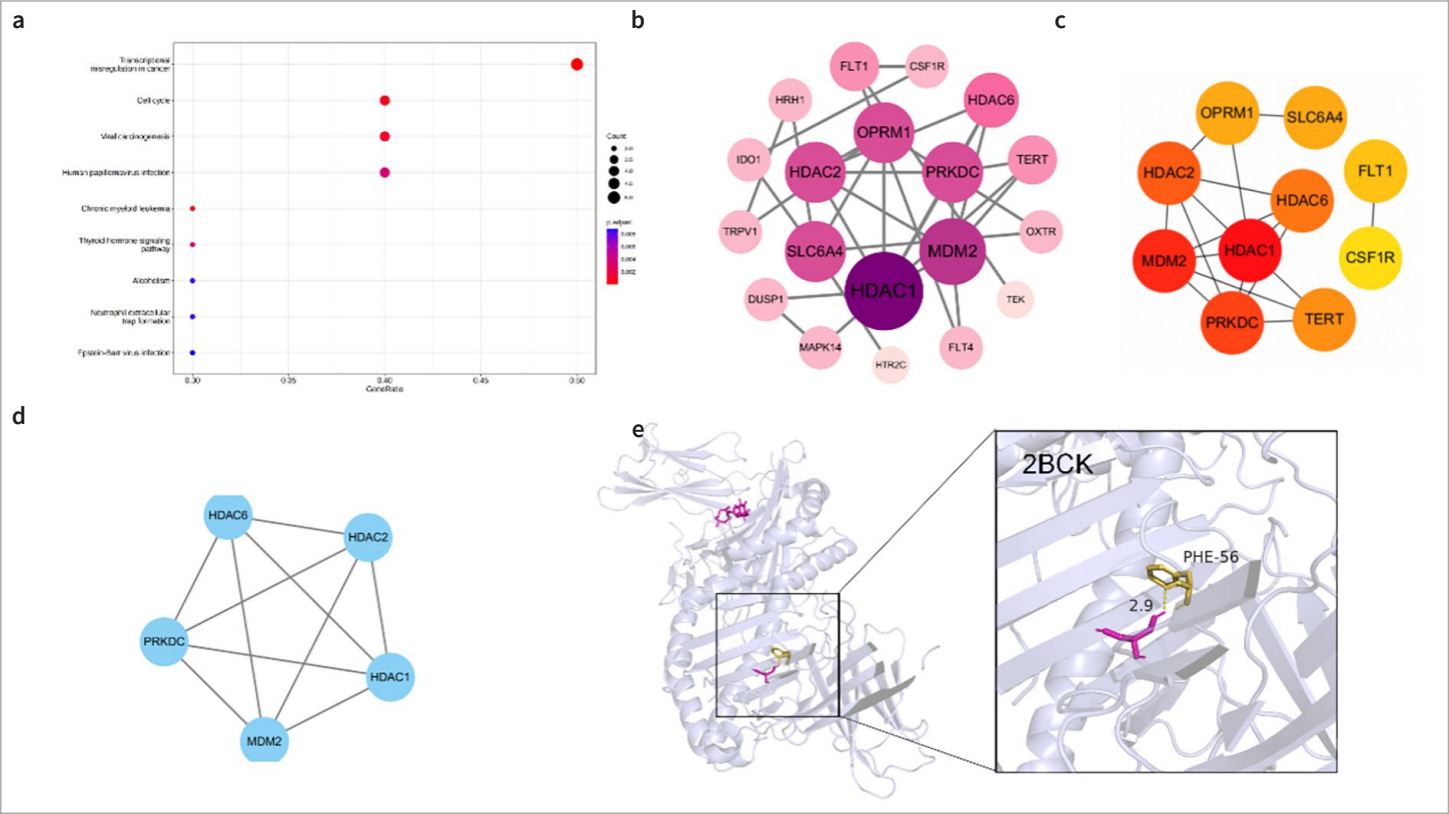
Network pharmacologic results of vitamin K treatment for periodontitis.(a) Vitamin K and periodontitis disease target KEGG enrichment analysis chart. (b) Interaction map for vitamin K and periodontitis disease targets. Arrange them in descending order of degree value and from deep to shallow. (c) Vitamin K and periodontitis Hubba gene disease targets. (d) Mcode classification of vitamin K and periodontitis disease targets. (e) Molecular docking results of vitamin K and periodontitis disease targets. The molecular docking results indicated the binding affinity between vitamin K and the core target protein (PDB:2BCK) and marked the key residues.

A total of 23 common genes were identified by screening 100 vitamin K-related targets and combined with 603 periodontitis mechanism gene targets. After the STRING platform was introduced, the PPI network diagram was constructed with these 23 genes (Fig 6b). In addition, the Molecular Complexity Detection (MCODE) algorithm identifies more tightly connected network components. Subsequently, 10 core targets with high probability were selected according to the DEGREE value: *HDAC1*, *MDM2*, *PRKDC*, *HDAC2*, *HDAC6*, *TERT*, *SLC6A4*, *OPRM1*, *FLT1*, *CSF1R* (Fig 6c). Molecular docking is performed. It is worth noting that, through molecular docking with 2BCK, it was found that vitamin E mainly interacted with the target at position 56, the PHE of 2BCK, and regulated its function (Fig 6e). The core targets were analysed using KEGG pathway enrichment (Fig 6a).

Our integrated approach, combining evidence synthesis and network pharmacology, has successfully delineated the multifaceted mechanisms through which vitamins A, C, D, E, and K may ameliorate periodontitis. The analysis identified several high-probability core targets (eg, *ESR1*, *PPARG*, *GSK3B*, *STAT3*) common to multiple vitamins. However, the true significance of these findings lies not merely in their identification but in their well-established biological functions within the pathogenesis of periodontitis. A critical interpretation of these targets reveals a convergent molecular theme: the modulation of hyperinflammatory responses and dysregulated bone metabolism.

For instance, the oestrogen receptor alpha (ESR1), a key target for vitamins A and D, is not only pivotal for bone mineral homeostasis but also a potent modulator of the immune response. In the context of periodontitis, impaired ESR1 signalling is associated with exacerbated production of pro-inflammatory cytokines (eg, IL-6) and increased osteoclast activity, contributing to accelerated alveolar bone loss. The interaction of vitamins with ESR1 suggests a plausible mechanism for their bone-protective and immunomodulatory effects, potentially mimicking some beneficial aspects of oestrogen signalling in the periodontium.

Similarly, the peroxisome proliferator-activated receptor gamma (PPARG), identified as a target for vitamins E and A, is a central regulator of macrophage polarisation and lipid metabolism. Its activation drives macrophages toward an anti-inflammatory (M2) phenotype, thereby dampening the chronic inflammatory response that drives tissue destruction in periodontitis. The enrichment of this target provides a novel mechanistic rationale for the anti-inflammatory properties of these vitamins, suggesting they may promote a pro-resolutive environment in periodontal tissues.

Furthermore, glycogen synthase kinase-3 beta (GSK3B), a common hub target, is a crucial kinase that promotes NF-κB activation and the subsequent synthesis of TNF-α, IL-1β, and other mediators central to the pathogenesis of periodontitis. The inhibition of GSK3B activity represents a powerful anti-inflammatory strategy. The prediction that multiple vitamins can interact with GSK3B underscores a shared pathway for mitigating the hyperinflammatory state that characterises progressive periodontitis.

Therefore, the functional aggregation of the predicted core targets into key pathological processes – rather than the identity of any single target – provides the strongest mechanistic support for the potential efficacy of vitamins in controlling the inflammatory and bone-destructive cycles of periodontitis.

#### CONCLUSIONS

##### Clinical Relevance

This study, through systematic integration of evidence and network pharmacological analysis, established the significant role of the vitamin family (especially vitamins D and C) in the prevention and treatment of periodontitis. They have transformed from mere essential nutrients into pleiotropic therapeutic tools for us to understand and manage the complex disease of periodontitis.

Vitamin D can enhance immune regulation and support alveolar bone preservation, potentiating the effects of conventional SRP. Assessment of serum vitamin D levels should be considered a routine part of periodontal evaluation, especially in patients with severe bone loss or non-responsive disease. Clinicians should advocate for adequate dietary intake and consider supplementation in patients with obvious deficiency, oxidative stress conditions (eg, smokers, diabetics), or those undergoing periodontal surgery. Its role in bolstering antioxidant defences and promoting soft tissue wound healing makes it a valuable, low-risk adjunct. Vitamin K also shows strong potential for supporting bone metabolism. Its co-supplementation with vitamin D may offer synergistic benefits for patients with comorbid osteoporosis or those at high risk of progressive bone loss. Vitamin A and vitamin E also play a crucial role in the antioxidant and immune aspects of periodontitis, but their clinical application may require more experimental verification.

However, it should still be noted that although vitamins show great potential in the adjuvant treatment of periodontitis, the potential adverse effects or toxicity brought about by high-dose supplementation (especially fat-soluble vitamins) must be vigilant. The therapeutic window for vitamins is not always broad. Supplementation beyond an individual’s tolerance limit may cause a series of health problems, thus going against the original intention of treatment. This risk mainly stems from the characteristic that fat-soluble vitamins (such as A, D, E, and K) are difficult to be rapidly excreted through the kidneys in the body and tend to accumulate in adipose tissue and the liver. Therefore, blindly pursuing high doses not only fails to enhance the therapeutic effect but may also disrupt the body’s inherent metabolic balance and trigger toxic reactions. Before recommending a supplementary plan, clinicians must follow the principle of ‘precision nutrition’ and fully assess the individual condition of the patient (Table 1).

**Table 1:** Key mechanisms and predicted molecular targets of vitamins in periodontitis

Vitamin	Key mechanisms	Key predicted targets*
Vitamin A	Immunomodulation (T-cell development) maintenance of epithelial barrier integrity regulation of bone metabolism	*ESR1*, *EGFR*, *MAPK1*, *GSK3B*, *AR*, *PPARG*
Vitamin C	Potent antioxidant (scavenges ROS) promotes collagen synthesis enhances immune response inhibits inflammatory markers	*STAT3*, *JUN*, *PTGS2*, *PRKCA*, *PPARG*
Vitamin D	Immunomodulation (inhibits pro-inflammatory cytokines, promotes antimicrobial peptides) promotes calcium/phosphorus absorption, regulates bone metabolism	*ESR1*, *AR*, *VDR*, *CYP27B1*, *TRPV1*
Vitamin E	Fat-soluble antioxidant anti-inflammatory	*AKT1*, *PTGS2*, *PPARG*, *ESR1*, *GSK3B*
Vitamin K	Activates osteocalcin inhibits vascular calcification inhibits pro-inflammatory cytokines	*HDAC1*, *MDM2*, *PRKDC*, *HDAC2*, *HDAC6*
*Predicted targets are derived from network pharmacology analysis (Hub genes).

##### Prospects and Innovations for Vitamin Treatment of Periodontitis

With the continuous exploration of the pathological mechanism of periodontitis and the biological function of vitamins in modern medicine, the multi-dimensional value of vitamins in the prevention and treatment system of periodontal diseases has become increasingly prominent. Recent studies have found that periodontitis is not only a local microbial infectious disease, but also closely related to host immune imbalance, oxidative stress and tissue repair ability. In this context, vitamins A, D, C, E, and vitamin K have shown potential advantages of multi-target intervention through their unique biological characteristics. The application prospect of vitamins in the prevention and treatment of periodontitis is gradually clear, which is mainly reflected in the following aspects:

###### Precision nutrition and personalised treatment

Building upon our network pharmacology findings that revealed distinct yet overlapping target profiles for each vitamin. Based on gene target analysis, biomarker detection can be used to evaluate the vitamin metabolism status and inflammation level of patients in the future, and a personalised supplement plan can be formulated. This approach moves beyond generic supplementation towards data-driven interventions based on an individual’s specific molecular pathology.

###### The value of prevention and early intervention

Vitamins, as an important part of the daily diet, have great potential in the primary prevention of periodontitis. Our systematic mapping of their multifaceted mechanisms – from immunomodulation (vitamins A, D) to antioxidant defence (vitamins C, E) and bone metabolism (vitamins D, K) – provides a strong scientific rationale for promoting vitamin-rich dietary patterns through public health education, combined with regular monitoring of serum vitamin levels, may significantly reduce morbidity in at-risk populations.

###### Synergies with existing therapies

Vitamin-assisted traditional treatments (eg, subgingival scaling for SRP) have shown synergistic effects. Future research may explore the combination of vitamins and probiotics, photodynamic therapy or immunomodulators to further improve periodontal tissue regeneration and inhibit pathogen colonisation.

##### Challenges and Limitations of Vitamin Therapy for Periodontitis

Still, the use of vitamins in the treatment of periodontitis is not without its challenges. As a micronutrient with multiple functions such as immune regulation, antioxidant and tissue repair, it has shown potential in regulating periodontal inflammation and bone metabolism imbalance, but the key bottleneck of clinical translation still needs to be broken. The significant challenges in its application include the following:

###### Individual differences and bioavailability

A patient’s genetic background, intestinal absorptive capacity, dietary habits, and comorbidities (eg, diabetes mellitus) may significantly affect the efficiency of vitamin metabolism. For example, vitamin D receptor (VDR) gene polymorphisms may cause some patients not to respond to complementary therapy. Furthermore, our network pharmacology predictions, while identifying potential targets, are based on generalised databases and cannot yet accurately predict these individual metabolic and pharmacodynamic responses; there is a gap between computational models and clinical reality.

###### Economic and compliance barriers

Long-term vitamin supplementation may increase the financial burden on patients, while complex medication regimens (eg, multiple daily doses or specific dietary requirements) may reduce adherence. In addition, the standardised production and quality control of vitamin supplements still need to be improved to avoid the impact of ingredient differences on efficacy.

###### Practical barriers to translation

How to translate the target discovery of basic research into clinically actionable intervention strategies may still require interdisciplinary collaboration. Crucially, the mechanistic insights and target predictions generated by computational methods, such as those presented in this study, remain hypothetical until they are rigorously validated through *in vitro* and *in vivo* experiments. This functional validation is a significant step before clinical application.

In summary, the prospects and challenges of vitamin therapy for periodontitis coexist. The molecular docking results, which suggest stable binding affinities between vitamins and core targets like *STAT3* and *ESR1*, provide a foundational hypothesis for structure-guided therapeutic development. This computational evidence can steer future research towards designing novel vitamin analogues or formulations with enhanced bioavailability and target specificity, potentially overcoming the limitations of natural vitamins. Ultimately, these in silico predictions must be translated through high-throughput screening assays and controlled clinical trials to validate their efficacy in modulating the identified pathways and improving periodontal outcomes in patients. In the future, it is necessary to break through the existing bottlenecks through multicentre clinical research, integration of advanced technologies (such as omics analysis and artificial intelligence), and cross-field collaboration, and finally realise the transformation from ‘laboratory evidence’ to ‘clinical practice’, so as to provide safer, more accurate and sustainable treatment strategies for periodontitis patients.
